# Prolonged Neuromuscular Blockade Following Succinylcholine Administration and the Clinical Importance of Family History: A Case Report

**DOI:** 10.7759/cureus.78814

**Published:** 2025-02-10

**Authors:** Gordon Hubbell, Stewart Slomowitz, Imani Thornton

**Affiliations:** 1 Anesthesiology, HCA Florida Westside Hospital, Plantation, USA

**Keywords:** anesthesia complications, neuromuscular blockers, neuromuscular depolarizing agents, pseudocholinesterase, pseudocholinesterase deficiency

## Abstract

When evaluating the etiology of delayed emergence from anesthesia, several differential diagnoses must be considered, with family history often being an overlooked factor. This case report highlights the clinical significance and diagnostic challenges of one such differential - pseudocholinesterase deficiency (PD). PD is a known defect in the pseudocholinesterase enzyme that may be either inherited or acquired. This deficiency impairs the metabolism of certain pharmacologic agents, including succinylcholine, a short-acting depolarizing neuromuscular blocking agent widely used in operating rooms to facilitate endotracheal intubation. As a result, affected individuals experience prolonged neuromuscular blockade, with recovery times varying significantly and initial diagnosis often proving difficult. Although not uncommon, PD is insidious, and most individuals with this deficiency remain undiagnosed until they or a family member experience prolonged emergence from anesthesia. Laboratory tests such as the dibucaine inhibition test can quantify the deficiency, as demonstrated in this case. This report describes a 90-year-old female who was found to have heterozygous atypical PD after experiencing significantly delayed recovery - lasting eight hours - following succinylcholine administration during hip arthroplasty in the lateral position. The diagnosis was made clinically through the process of elimination and later confirmed by the dibucaine inhibition test. Postoperatively, it was revealed that several of the patient’s family members had previously experienced delayed emergence from anesthesia, suggesting an inherited form of PD that was previously unknown to the patient. This case underscores the importance of considering PD in the differential diagnosis of prolonged emergence from anesthesia, highlights the value of a thorough family history, and emphasizes the need for greater awareness among clinicians to prevent life-threatening adverse events.

## Introduction

Delayed emergence from anesthesia is a significant and often challenging complication that can occur during or after surgical procedures. It has a wide range of potential causes, including residual anesthetic effects, metabolic disturbances such as hypercarbia or hypoglycemia, central nervous system dysfunction, and pharmacologic interactions [1,2]. One often-overlooked cause is pseudocholinesterase deficiency (PD), a genetic or acquired condition in which a deficiency or malfunction of the pseudocholinesterase enzyme (also known as butyrylcholinesterase or plasma cholinesterase) slows the breakdown of certain medications. These include local ester anesthetics, mivacurium, and the commonly used neuromuscular blocking agent succinylcholine [2].

Succinylcholine is a depolarizing neuromuscular blocking agent that is typically broken down rapidly by pseudocholinesterase into its inactive metabolites, succinylmonocholine and choline [2]. It is widely used to induce temporary paralysis for intubation; however, in individuals with PD, its effects can persist far longer than expected, leading to prolonged respiratory paralysis and difficulty in weaning from mechanical ventilation.

PD is inherited in an autosomal recessive manner and manifests in two primary forms: homozygous atypical, characterized by near-complete enzyme deficiency, and heterozygous atypical, associated with partial enzyme deficiency. The estimated prevalence of PD is between 1 in 3,200 and 1 in 5,000 individuals [3]. PD can also be acquired due to conditions such as liver disease, burns, advanced age, malnutrition, and pregnancy [4].

The dibucaine number, a laboratory test, aids in diagnosing PD by measuring the degree to which the enzyme is inhibited by the local ester dibucaine [3]. Unfortunately, many individuals remain undiagnosed until they experience delayed emergence from anesthesia, as there are no overt symptoms beforehand. A thorough preoperative history, including questions about family members’ anesthesia-related complications, can serve as a critical clue for identifying an inherited form of PD. While reports of anesthesia complications may sometimes be subjective or nonspecific, paying close attention to a history of difficulty waking from anesthesia or unusual sensitivity to anesthetic agents can help prevent serious adverse events.

Additionally, even patients who have experienced prolonged emergence due to PD may not have undergone a quantitative blood test, such as the dibucaine inhibition test. As a result, they may remain unaware of their deficiency, placing themselves or their family members at risk for future anesthesia-related complications.

Early recognition of PD enables clinicians to tailor anesthetic management and mitigate potentially severe complications, such as prolonged mechanical ventilation and intensive care unit admission. This case report highlights the importance of considering PD in the differential diagnosis of prolonged emergence from anesthesia, particularly in patients with a family history of anesthesia-related complications.

## Case presentation

The patient was a 90-year-old Caucasian female, classified as ASA 3, weighing 45 kg, standing 5 ft tall, with a BMI of 19. She was scheduled for an elective left hip arthroplasty due to a left hip fracture sustained from a mechanical fall while walking out her front door. Her past medical history included hypertension, hyperlipidemia, paroxysmal atrial fibrillation, a pulmonary embolism more than six months prior, tobacco abuse, and osteoarthritis. She had no known drug allergies and had previously undergone an appendectomy over 20 years ago without reported complications. Her home medications included Eliquis, which had been discontinued three days before surgery during her hospital stay, as well as amlodipine and metoprolol. She was a reliable historian who reported being active at home without requiring assistive devices such as a cane or walker, and she had strong family support. Notably, she denied any previous anesthesia complications; however, her family members were not present at the time of questioning. Preoperatively, she was in normal sinus rhythm, normotensive, and maintaining adequate oxygen saturation on room air, although she complained of severe left hip pain. Airway examination revealed a Mallampati II classification with some limitation in neck extension, and she had top and bottom dentures. Preoperative lab results were largely unremarkable except for a slightly elevated white blood cell count (Table 1).

**Table 1:** Preoperative laboratory results

Lab test	Result
White blood cell count	13.5 (High)
Hemoglobin	12.4
Hematocrit	38.2
Platelets	178
Sodium	136
Potassium	4.3
Chloride	111 (High)
Blood urea nitrogen	37 (High)
Creatinine	1.12
Glomerular filtration rate	51
Glucose	117
Calcium	8.8
Aspartate transaminase	36
Alanine transaminase	20
Albumin	4.4
Magnesium	2
Phosphorus	3.3
Prothrombin time	11.7
International normalized ratio	1
Partial thromboplastin time	25

To optimize pain control, the patient received a pericapsular nerve group (PENG) block with 20 mL of 0.5% ropivacaine, which targets branches of the femoral, obturator, and accessory obturator nerves. Due to the planned right lateral positioning with the left side up, endotracheal intubation was preferred over a laryngeal mask airway (LMA) for better airway protection. Anesthesia was induced with 100 mg of succinylcholine, 100 mg of propofol, and 25 mcg of fentanyl. Endotracheal intubation was successfully performed using a Mac 3 blade and a 7.0 mm endotracheal tube without difficulty. Anesthesia was maintained with sevoflurane for the two-hour procedure, and the patient was placed on a pressure-control volume-guarantee ventilation mode, which regulated respiratory rate, pressure, and tidal volume. No additional doses of neuromuscular blockers (NMBs) or opioids were administered intraoperatively. Temperature monitoring and bispectral index (BIS) monitoring, used to assess the depth of anesthesia, were continuously utilized. The patient remained hemodynamically stable throughout the procedure, requiring no additional analgesia, as the PENG block provided effective pain relief.

At the conclusion of the surgery, despite discontinuation of volatile anesthetics for over 45 minutes, the patient exhibited no signs of respiratory recovery. She remained apneic and unresponsive to noxious stimuli, with an absent gag reflex and no spontaneous ventilation. Her BIS value increased from the 50s intraoperatively to the 90s, indicating consciousness, while tachycardia and hypertension further suggested awareness. Stroke was initially considered; however, intact pupillary responses made this diagnosis less likely. Neuromuscular monitoring revealed zero twitches in response to stimulation at multiple sites, including the ulnar and facial nerves, raising concern for prolonged neuromuscular blockade. Laboratory investigations, including blood gas analysis and electrolyte levels, were largely unremarkable, ruling out metabolic causes of delayed emergence (Table 2, Table 3). Naloxone (0.4 mg IV) was administered to exclude opioid overdose but provided no improvement. Additionally, medication administration records were reviewed, confirming that no long-acting NMB had been mistakenly given. Based on clinical findings and through the process of elimination, PD was suspected as the likely cause due to the use of succinylcholine. A cerebrovascular accident was also considered, and the patient was re-sedated with propofol before undergoing a CT scan of the brain. The results were unremarkable, and she was subsequently transferred to the ICU for mechanical ventilation and observation.

**Table 2:** Postoperative arterial blood gas results

Arterial blood gas parameter	Result
pH	7.35
pCO₂	38.5
pO₂ on 100% FiO₂	273.50 (High)
PO₂/FiO₂ ratio	273.5
HCO₃	21 (Low)
Base excess	-4.10 (Low)
Oxyhemoglobin	98.4
Carboxyhemoglobin	1.5
Methemoglobin	0.1
FiO₂	100
Ventilation mode	Pressure-controlled volume-guaranteed
Positive end-expiratory pressure	5

**Table 3:** Interpretation of dibucaine inhibition test results

% Inhibition	Reference range
Normal	70-90
Heterozygotes	30-70
Homozygotes	0-30

At this time, the patient’s family was contacted, revealing that her son had previously experienced delayed emergence after anesthesia, although he had never received a formal diagnosis. Approximately eight hours after succinylcholine administration, the patient regained spontaneous ventilation and was extubated in stable condition with full respiratory recovery. Interestingly, she later recalled experiencing a brief sensation of being “frozen”; however, due to her re-sedation, it was unclear at which point this had occurred. Further discussion with the family revealed additional relatives with unexplained difficulty awakening from anesthesia, although no formal diagnosis of PD had ever been established. A dibucaine inhibition test was ordered, returning a result of 41 - consistent with a heterozygous atypical variant of PD (Table 4, Table 5). The patient and her family were counseled on the inherited nature of this condition and the potential implications for relatives. She was discharged home safely within 24 hours.

**Table 4:** Postoperative laboratory results

Lab test	Result
White blood cell count	9.6
Hemoglobin	12.3
Hematocrit	39.2
Platelets	174
Sodium	134
Potassium	4
Chloride	108
Blood urea nitrogen	36 (High)
Creatinine	1.22 (High)
Glomerular filtration rate	42
Glucose	125
Calcium	8.4

**Table 5:** Patient’s dibucaine inhibition test results

Test	Result
Dibucaine number	41 (Low)

## Discussion

This case highlights a clinically significant instance of prolonged neuromuscular blockade due to heterozygous atypical PD. NMBs play a crucial role in anesthesia and critical care by inducing muscle relaxation, facilitating intubation, and optimizing surgical conditions. These agents are classified into depolarizing and nondepolarizing types based on their mechanism of action.

Succinylcholine is the primary depolarizing NMB. It functions as an acetylcholine receptor agonist, causing continuous depolarization of the neuromuscular junction, which results in muscle paralysis [5]. In contrast, nondepolarizing NMBs act as competitive antagonists at the acetylcholine receptor, preventing acetylcholine from binding and thereby inhibiting muscle contraction. These agents are further categorized into steroidal compounds (e.g., rocuronium and vecuronium) and benzylisoquinoline compounds (e.g., atracurium and cisatracurium) [5].

Succinylcholine is widely used as an induction agent for endotracheal intubation due to its rapid onset of approximately 45 seconds and short duration of action, typically lasting 10 to 15 minutes. This makes it particularly useful in cases where brief muscle paralysis is needed, such as managing a potentially difficult airway where prolonged neuromuscular blockade is undesirable [6].

However, succinylcholine is associated with several potential adverse effects, including muscle pain, hyperkalemia, increased intraocular and intragastric pressure, sinus bradycardia, and malignant hyperthermia. Nondepolarizing agents also have side effects, such as histamine release, which can lead to hypotension and tachycardia [5].

Succinylcholine administration can result in two distinct types of neuromuscular blockade: Phase I and Phase II. Understanding the differences between these blocks is essential for appropriate clinical management.

In a Phase I block, succinylcholine acts as an agonist at nicotinic acetylcholine receptors in the neuromuscular junction, causing continuous depolarization of the motor end plate. This persistent depolarization prevents repolarization, leading to muscle paralysis. Clinically, a Phase I block is characterized by muscle fasciculations followed by flaccid paralysis. Peripheral nerve stimulation reveals no fade with train-of-four (TOF) stimulation and no post-tetanic potentiation. The block is typically short-lived, lasting four to six minutes after a single IV dose due to rapid hydrolysis by plasma cholinesterase [7].

A Phase II block can occur with prolonged or repeated administration of succinylcholine. This phase resembles a nondepolarizing blockade, where the neuromuscular junction becomes desensitized to acetylcholine, and ion channels close, preventing further depolarization. Unlike a Phase I block, Phase II is characterized by a fade in response to TOF stimulation and post-tetanic potentiation, similar to nondepolarizing NMBs. The duration of a Phase II block is variable and can be significantly prolonged, particularly in patients with atypical plasma cholinesterase or those receiving high doses of succinylcholine [7].

Regarding reversal, a Phase I block should not be reversed with anticholinesterase agents like neostigmine, as this can prolong the blockade. In contrast, a Phase II block may sometimes be reversed with neostigmine, but this requires careful diagnosis using peripheral nerve stimulation to confirm Phase II characteristics before attempting reversal [7].

In summary, a Phase I block is a depolarizing blockade with no fade on TOF stimulation, while a Phase II block mimics a nondepolarizing blockade, exhibiting fade and post-tetanic potentiation. Proper identification and management of these blocks are crucial for ensuring patient safety and optimal use of succinylcholine.

In this case, the patient’s PD resulted in a drastically prolonged Phase I block, which would not have benefited from reversal with an anticholinesterase agent such as neostigmine. Her paralysis lasted approximately eight hours before she could be safely extubated, raising the important question of why succinylcholine was used in the first place. Alternative induction agents such as rocuronium, a nondepolarizing NMB, could have been used instead to facilitate intubation. Although rocuronium has a longer duration of action, it would have prevented this complication, as it is not metabolized by pseudocholinesterase. Additionally, the reversal agent sugammadex could have allowed rapid and efficient neuromuscular blockade reversal by directly binding rocuronium and vecuronium. Another consideration was whether intubation could have been avoided entirely with the use of an LMA, eliminating the need for paralytics and preventing prolonged intubation.

Several unique circumstances influenced the decision to proceed with endotracheal intubation and the use of succinylcholine over alternative options. The procedure was performed in the lateral position, meaning the patient’s body, including the head, would be turned to the side. While an LMA can be placed in this position, it is generally considered a less secure airway with an increased risk of displacement compared to an endotracheal tube. The patient was also edentulous, which may have further complicated LMA placement and stability, increasing the risk of dislodgement. Given her advanced age and history of pulmonary embolism, securing the airway was a primary concern to prevent intraoperative and postoperative complications. Succinylcholine was selected over rocuronium primarily to avoid prolonged paralysis and reduce the risk of postoperative respiratory weakness. Additionally, sugammadex was not readily available at this facility, further influencing the decision to use a short-acting paralytic instead of a longer-acting agent like rocuronium.

The patient’s history of a previous appendectomy under general anesthesia without complications raises the question of whether her PD was acquired rather than inherited. However, it is possible that she did not receive succinylcholine during her prior surgery. The fact that at least two of her family members reported a similar sensitivity to anesthesia strongly suggests an inherited form of PD.

The dibucaine inhibition test played a crucial role in confirming the diagnosis. This test measures the percentage of pseudocholinesterase enzyme activity inhibited by dibucaine HCl, an amide local anesthetic [3]. A dibucaine number below 70 is diagnostic of PD, with values between 30 and 70 typically seen in heterozygous patients and below 30 in homozygous individuals (Table 3) [5]. Acquired PD has been associated with conditions such as insulin resistance, chronic infections, renal disease, liver disease, malignancy, major burns, malnutrition, and pregnancy [5]. Certain medications, including cyclophosphamide, oral contraceptives, monoamine oxidase inhibitors, and glucocorticoids, have also been linked to acquired enzyme deficiency [5].

In this case, prolonged emergence was not suspected until the end of the procedure when the patient failed to regain spontaneous ventilation and showed no response to neuromuscular stimulation. TOF monitoring, a tool used to assess neuromuscular blockade, has limitations, including intermittent rather than continuous monitoring and potential inaccuracies, which may delay recognition of PD [7]. Additionally, the patient remained on the ventilation mode of pressure-controlled volume-guaranteed throughout the case, preventing earlier detection. Spontaneous intraoperative ventilation could have eliminated suspicion of PD earlier.

Genetically, PD is inherited in an autosomal recessive pattern and affects approximately 1 in 3,200 to 1 in 5,000 individuals, with a higher prevalence among Persian Jewish and Native Alaskan populations [5]. Given the family history, genetic counseling could have facilitated early diagnosis and awareness among relatives. While heterozygous patients typically experience a milder and delayed response to succinylcholine, they may still face significant complications, underscoring the importance of preoperative risk assessment.

Succinylcholine has a unique mechanism of action, and currently, no pharmacological agents can directly reverse its effects. The primary approach to managing succinylcholine-induced paralysis is supportive care, including mechanical ventilation, until the drug is metabolized by plasma cholinesterase. Research has explored recombinant butyrylcholinesterase as a potential reversal agent for succinylcholine-induced apnea, particularly in patients with enzyme deficiency. Studies have demonstrated that recombinant butyrylcholinesterase can hydrolyze succinylcholine in vitro and reverse its effects in animal models, suggesting a potential therapeutic application in the future [8]. However, as of now, no approved medications exist to reverse a Phase I block caused by succinylcholine, making supportive care the cornerstone of management.

This case underscores the critical importance of a thorough family history in preoperative evaluations. While the patient initially denied any previous surgical complications, two family members later reported experiencing delayed emergence from anesthesia. This highlights the necessity of asking not only about the patient’s own surgical history but also about any family history of anesthesia-related complications, as this information could help prevent adverse events.

Several key lessons emerge from this case, particularly in the management of succinylcholine-induced neuromuscular blockade. A comprehensive preoperative history, including a detailed family history, is crucial, as PD often remains undiagnosed until a patient experiences prolonged paralysis following succinylcholine administration. Continuous intraoperative monitoring of the TOF ratio could have allowed for earlier detection of prolonged neuromuscular blockade, aiding in the anticipation of supportive care needs and reducing the risk of intraoperative awareness.

Recognition of the patient’s genetic variance in the butyrylcholinesterase gene could have prevented the administration of succinylcholine altogether. Genetic testing in individuals with a family history of PD or anesthesia-related complications could serve as a valuable tool in preventing unexpected prolonged paralysis. Management of a prolonged blockade primarily involves supportive care, including mechanical ventilation, until spontaneous recovery occurs. The use of anticholinesterase agents like neostigmine is not recommended for a Phase I block but may be considered for a Phase II block after confirmation via neuromuscular monitoring.

Proper documentation and communication are essential to ensure that future anesthetic plans account for this condition. Patients diagnosed with PD should be thoroughly educated about their condition and the implications for future anesthesia. Additionally, family members may benefit from genetic counseling and testing to identify at-risk individuals and prevent similar complications in the future.

## Conclusions

This case underscores the importance of recognizing PD as a potential cause of delayed emergence from anesthesia, particularly following the administration of succinylcholine. It also highlights the critical role of family history in identifying anesthesia-related complications. The diagnosis in this case was established through a combination of neuromuscular monitoring, process of elimination, and confirmation via the dibucaine inhibition test. Clinicians should maintain a high index of suspicion for PD, especially in patients with a family history of delayed emergence, as the condition often remains undiagnosed until an adverse event occurs. Preoperative screening, including genetic testing in high-risk individuals, may aid in early detection, while the use of alternative neuromuscular blocking agents such as rocuronium can help mitigate the risks associated with prolonged paralysis. However, these diagnostic and preventive tools may not always be readily available.

Given the potential for PD to significantly impact patient outcomes, clinicians should always consider this condition when administering succinylcholine, particularly in the presence of a suggestive family history. This case illustrates the challenges of diagnosing PD and emphasizes the need for heightened awareness and careful perioperative management in susceptible patients. Key takeaways from this case include the importance of a thorough preoperative assessment, continuous neuromuscular monitoring, recognition of genetic variants, appropriate management of prolonged blockade, and effective postoperative care and communication. These insights are essential for optimizing the safe and effective management of succinylcholine-induced neuromuscular blockade in patients with PD.
